# Centriole assembly and the role of Mps1: defensible or dispensable?

**DOI:** 10.1186/1747-1028-6-9

**Published:** 2011-04-14

**Authors:** Amanda N Pike, Harold A Fisk

**Affiliations:** 1 Department of Molecular Genetics, The Ohio State University, 484 W. 12th Avenue, Columbus OH 43210-1292, USA

## Abstract

The Mps1 protein kinase is an intriguing and controversial player in centriole assembly. Originally shown to control duplication of the budding yeast spindle pole body, Mps1 is present in eukaryotes from yeast to humans, the nematode *C. elegans *being a notable exception, and has also been shown to regulate the spindle checkpoint and an increasing number of cellular functions relating to genomic stability. While its function in the spindle checkpoint appears to be both universally conserved and essential in most organisms, conservation of its originally described function in spindle pole duplication has proven controversial, and it is less clear whether Mps1 is essential for centrosome duplication outside of budding yeast. Recent studies of Mps1 have identified at least two distinct functions for Mps1 in centriole assembly, while simultaneously supporting the notion that Mps1 is dispensable for the process. However, the fact that at least one centrosomal substrate of Mps1 is conserved from yeast to humans down to the phosphorylation site, combined with evidence demonstrating the exquisite control exerted over centrosomal Mps1 levels suggest that the notion of being essential may not be the most important of distinctions.

## I. The Centrosome

In proliferating cells, organization of a bipolar mitotic spindle is facilitated by the presence of two mature centrosomes, each of which contains a pair of centrioles. Accordingly, the single centrosome that proliferating cells inherit must be precisely duplicated exactly once prior to mitosis [1]. The proper structure and function of centrosomes is dependent upon the strict doubling of existing centrioles [2], making the molecular mechanisms underlying the centriole assembly cycle of particular importance in the maintenance of genomic integrity.

**Figure 1:**
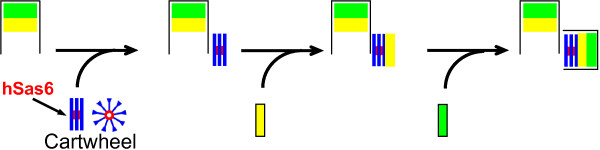
**Centriole assembly as a modular process**. Centriole assembly in human cells proceeds through a pathway analogous to that described in *C. elegans*, but requires several additional proteins not present in worms, and proceeds through a cartwheel template as opposed to a central tube. Centrioles are not templates *per se*, but rather provide a surface for the assembly of cartwheels, depicted as a hub composed of hSas6 (red) and 9 symmetrical spokes (blue). We propose that cartwheels then serve as a platform onto which additional centriole modules are assembled in a proximal to distal, or "bottom-up" fashion. Yellow and green rectangles are used to depict proximal (yellow) and distal (green) centriole modules. The frame surrounding these modules is meant to depict maturation into the final structure rather than centriolar microtubules.

The canonical centrosome assembly pathway results in the construction of a single procentriole at a site adjacent to each existing centriole (Figure 1). Much of what is known about centrosome duplication is derived from studies in model organisms [3,4]. A powerful proteomic and comparative genomic analysis in green algae led to the characterization of 18 core proteins that form the Proteome of Centrioles, called the Poc proteins [5], and genome-wide RNAi screens in *C. elegans *identified five essential centriole biogenesis proteins, SPD-2, ZYG-1, SAS-4, SAS-5, and SAS-6 [6,7]. Live cell imaging of worm embryos placed these proteins into an ordered assembly pathway: pro-centriole formation is initiated by the recruitment of SPD-2 to an existing centriole, SPD-2 leads to recruitment of the ZYG-1 protein kinase, which in turn recruits a complex containing SAS-5 and SAS-6 that promotes the formation of a central tube that determines basic centriole structure, followed by SAS-4 that facilitates the assembly of microtubules and mediates pro-centriole elongation [6-11]. The procentriole formation pathway delineated in *C. elegans *represents a core centriole assembly program that is conserved in organisms as distinct as *T. thermophila *[12], *D. melanogaster *[13] and *H. sapiens *[14-16]. The past year has seen an explosion in our understanding of the canonical centriole assembly pathway in humans. The human SPD-2 orthologue Cep192 is required for both centriole biogenesis and centrosome maturation, binds to Plk4, and is required for Plk4-dependent centriole overproduction [17], suggesting that it might function analogously to SPD-2. However, Plk4 recruitment also requires Cep152, the human orthologue of *D. melanogaster *asterless [18,19]. Recruitment of Plk4, the distant relative and presumptive functional counterpart to ZYG-1 [15,16,20], is then followed by that of hSas6 [14,15] and the human orthologue of Sas-4, CPAP/CENP-J [21-23]. Several additional proteins are recruited during S and G2 to promote procentriole assembly and elongation, including Cep135 [15,24], γ-tubulin, and CP110 [15,25]. However, centriole assembly in human cells involves many additional proteins not found in worms, including δ- and ε- tubulins [26,27], Mps1 [28-32], Centrin 2 (Cetn2) [28,33], hPoc5 [34], and Cep76 [35], among others.

It was previously thought that mother centrioles serve as a template for the assembly of procentrioles, in analogy to DNA replication. This has largely been disproven [36], and perhaps a more accurate description is that a centriolar template is assembled at a site adjacent to an existing centriole. In worms this template is a central tube formed by the SAS5/SAS6 complex [7], while in vertebrates and many other organisms it is a structure called the cartwheel [37]; both structures serve as a platform around which procentrioles are assembled, and Sas-6 forms the central hub responsible for nine-fold symmetry [38]. Once assembled, procentrioles undergo elongation through S and G2, but remain attached to the proximal end of their mother until mitosis, when they become physically disengaged [39]. Centriole assembly would thus appear to be a modular process, wherein centrioles are built from the bottom up (Figure 1); existing centrioles provide a surface for the assembly of a cartwheel, which serves as platform onto which successive modules are assembled in a proximal to distal fashion. In support of this modular nature of centriole assembly, both hPoc5 and Cetn2 appear to be dispensable for the initiation of centriole assembly, but depletion of hPoc5 leads to production of centrioles that lack distal elements [34], and overexpression of Cetn2 can organize a subset of distal centriole proteins at sites other than cartwheels [28]. Two of the key players in this core centriole assembly pathway, Plk4 [40-42] and hSas6 [14] are regulated by degradation. The result of this regulation is that hSas6 can only be assembled into procentrioles once per cell cycle, and centriolar hSas6 is degraded during mitosis. As a consequence, while cells have either two (G1) or four (S, G2, M) centrioles, they have zero (G1 or M) or two (S and G2) hSas6 foci.

The presence of extra centrosomes leads to the formation of extra spindle poles and multipolar spindles [4]. These extra centrosomes perturb the proper connection of chromosomes to opposite spindle poles, resulting in chromosome mis-segregation and aneuploidy, even when the extra centrosomes cluster to produce pseudo-bipolar spindles [43,44]. Indeed, excess centrosomes have recently been shown to generate aneuploidy, and genetic instability driven by the presence of extra centrosomes is thought to be an early event in prostate and breast tumor progression [43-46]. The exact mechanisms responsible for restricting centrosome duplication remain to be elucidated, but overexpression of Cep76 can suppress centrosome re-duplication [35], while overexpression of several centriole assembly factors such Mps1 [31,47], Plk4 [15], hSas6 [14], or Cetn2 [28] can generate extra centrioles, indicating the importance of the strict regulation of centriole assembly factors throughout the cell cycle. This review aims to address recent advances in our understanding of the role of Mps1 in centriole assembly, as well as the controversy surrounding the function of centrosomal Mps1, and the future challenges for its understanding.

## II. An Introduction to Mps1

### Mps1, the p53 of the cytoskeleton?

Mps1 is a dual specificity protein kinase conserved from yeast to mammals [48]. Originally identified for its role in spindle pole body (SPB) duplication in budding yeast, Mps1 was named for the phenotype observed in mutant cells, which fail in SPB duplication and form **M**ono**p**olar **s**pindles upon entering mitosis [49]. Mps1 was subsequently shown to regulate the mitotic spindle assembly checkpoint (SAC) [50,51], and has since been found to function in a wide range of cellular processes in diverse organisms including hypoxia-induced mitotic arrest [52] and the meiotic assembly checkpoint in flies [53], and tissue regeneration in fish [54]. In human cells reported hMps1 functions include centrosome duplication [28-32], the SAC [55], SMAD signaling [56], the p53-dependent post-mitotic checkpoint [57], the Chk2-dependent DNA damage response [58], and cytokinesis [32]. The most recently documented function for Mps1 ties it to the actin cytoskeleton through a newly identified binding partner Mip1, a novel component of the actin cytoskeleton that regulates the interaction of the mitotic spindle with the cell cortex [59]. While each of its described functions is relevant to the control of genomic integrity, it is not clear to what extent each function of Mps1 is conserved. Regarding its originally defined functions from yeast, there is wide agreement that the SAC function of Mps1 is universally conserved among eukaryotes [55] but reports differ regarding a role for Mps1 at centrosomes [30,32,60,61]. Though the list of Mps1 functions continues to expand, this review will concentrate on its controversial role in centrosome duplication.

### From yeast to house apes

**Figure 2:**
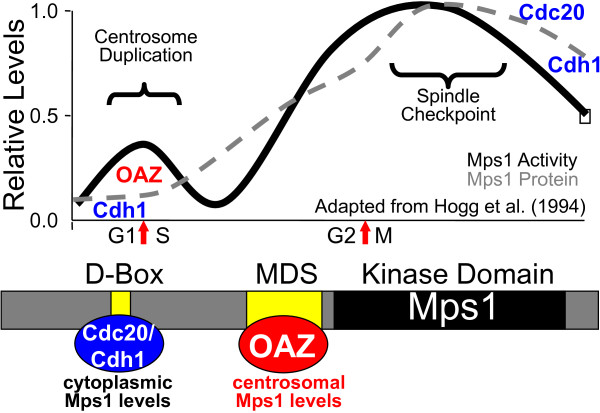
**Cell cycle profile of Mps1 and its control by degradation**. The top panel shows the cell cycle profile of hMps1 protein levels (broken grey line) and protein kinase activity (solid black line), adapted from the data of Hogg et al., 1994 [64]. After a sharp peak of hMps1 activity at G1/S that is not accompanied by a rise in whole cell protein levels, both protein and activity peak in mitosis. After completing its function in the spindle checkpoint, Mps1 is targeted for degradation at mitotic exit by both Cdc20- and Cdh1-associated APC/C complexes through the hMps1 D-box. Cdh1-dependent hMps1 degradation keeps cytoplasmic hMps1 levels low in G1, while OAZ targets the centrosomal pool of Mps1 for degradation through the MDS. Phosphorylation of T468 within the MDS transiently suppresses OAZ-mediated degradation, allowing accumulation of a centrosomal pool coincident with centrosome duplication. The lower panel shows a schematic of the 853 amino acid hMps1 protein indicating the positions of the D-box (amino acids 256-263) and MDS (amino acids 420-507) in yellow as the binding sites for Cdh1/Cdc20 (blue) and OAZ (red), respectively, and the kinase domain in black.

SPB duplication is well recognized as a model for centrosome duplication [3], and once vertebrate orthologues of Mps1 (originally cloned as Esk in mice [62] and TTK [63,64] or PYT [65] in humans, referred to hereafter generally as Mps1 or explicitly as mMps1 or hMps1, respectively) were recognized as such it was anticipated that some aspect of Mps1 function in SPB duplication might be conserved in vertebrates. mMps1 and hMps1 can autophosphorylate on tyrosines, and both were identified through screens for receptor tyrosine kinases. However, initial studies demonstrated them to be cell cycle regulated dual-specificity kinases, and suggested that both translational and post-translational inputs controlled their cell cycle profile [62,63,65]. Notably, while the levels of hMps1 message, protein, and kinase activity peaked during mitosis, there is a sharp peak of hMps1 kinase activity at the G1/S boundary that is not accompanied by a significant change in hMps1 protein levels [64] (Figure 2). Thus, hMps1 shows a sharp peak of modest total activity but high specific activity at G1/S that is coincident with centrosome duplication, and a second much larger peak of activity at G2/M coincident with the SAC.

### Mps1, of mice and men

The first functional studies of vertebrate Mps1 examined the mouse [66] and frog [67] enzymes. Both endogenous mMps1 and GFP-mMps1 localized to centrosomes and kinetochores in NIH 3T3 cells [66]. Overexpression of mMps1 caused centrosome re-duplication, while the catalytically inactive kinase dead (KD) version mMps1-KD blocked centrosome duplication, suggesting that mMps1 regulated centrosome duplication [66]. Concurrently, the *X. laevis *Mps1 was shown to be present at kinetochores where it is required for SAC function [67]. Accordingly, these initial studies supported the idea that the functions of Mps1 described in yeast were conserved in vertebrates. However, the first functional study of hMps1 cast doubt on this suggestion, as it failed to find hMps1 at centrosomes, or to reveal any defect in centrosome duplication after the overexpression of hMps1 (hMps1 or hMps1KD) or its siRNA-mediated depletion [61]. Rather, the only phenotype reported in that study was the failure of hMps1-depleted cells to arrest in mitosis in response to the microtubule poison nocodazole [61].

There were important experimental differences between the original mMps1 and hMps1 studies, and when used with timing and expression levels similar to the mMps1 study, hMps1KD and wild type hMps1 were subsequently shown to attenuate centrosome duplication and accelerate centrosome re-duplication, respectively, in human cells [30,32]. In addition, other groups have subsequently observed transgenic hMps1 at centrosomes [30,68], and at least four hMps1 antibodies (three different rabbit polyclonal, and one mouse monoclonal) have subsequently been shown to stain centrosomes [29,32,68,69] (although the vast majority of hMps1 antibodies do not). Notably, a recent study that identified activating phosphorylation sites within hMps1 not only found active Mps1 at centrosomes, but also showed that it interacts with γ-tubulin [69]. Moreover, the siRNA-mediated depletion of hMps1 can attenuate centrosome duplication [32]. Titration of hMps1-specific siRNAs suggested that centrosome duplication requires significantly less Mps1 than the SAC, and that not even mitotic catastrophe was indicative of complete hMps1 depletion [32]. Indeed, this finding is consistent with the very low levels of hMps1 at G1/S observed by Hogg et al [64] (Figure 2). Interestingly, these initial functional studies of hMps1 highlighted a difference between mouse and human cells; while moderate overexpression of wild type Mps1 is sufficient to drive centrosome re-duplication in mouse cells [66], even gross overexpression of wild type Mps1 is not sufficient do so in human cells [32,61], a difference that is due to cell type and not the source of enzyme (unpublished observations). Our subsequent explorations have suggested that this difference is due to the regulation of Cdk2 activity between mice and humans, although this has not been explicitly tested.

## III. Exquisite control over centrosomal Mps1 levels

The G1 to S transition in vertebrate cells marks "the point of no return" in the cell cycle, beyond which the cell is committed to division, and Hogg et al. showed a dramatic peak of hMps1 kinase activity at G1/S with little associated change in total protein levels [64] (Figure 2). This suggests that the function of Mps1 in centrosome duplication is controlled by a posttranslational mechanism, and the initial mMps1 study showed that Cdk2 suppresses the proteasome-mediated degradation of mMps1 [66]. Overexpression of the Cdk2 partner cyclin A causes centrosome reduplication in human cells [70], and further studies suggested that cyclin A-associated Cdk2 (Cdk2/A) specifically regulates the degradation of a centrosomal pool of hMps1; while cyclin A overexpression increased the level of hMps1 at centrosomes by ~2.5-fold, modulation of Cdk2 or the proteasome had little effect on the whole cell levels of Mps1 [31]. Amino acids 420-507 that are encoded by hMPS1 exons 12 and 13 contain an **M**ps1 **D**egradation **S**ignal (MDS, see Figure 2). The MDS is responsible for the proteasome-dependent removal of hMps1 from centrosomes in the absence of Cdk2 activity, and a single site within the MDS, T468, is phosphorylated by Cdk2/A [31]. The non-phosphorylatable GFP-hMps1^T468A ^can accumulate in the cytoplasm but is constitutively removed from centrosomes in a proteasome-dependent manner [31]. GFP-hMps1^T468A ^cannot substitute for the function of endogenous hMps1 in centrosome duplication, demonstrating that it is the centrosomal pool of hMps1 that is relevant to its function in centrosome duplication [31]. In contrast, the GFP-hMps1^T468D ^and GFP-hMps1^T468E ^mutants that mimic T468 phosphorylation, and GFP-hMps1^Δ12/13 ^that lacks the MDS, can accumulate at centrosomes in the absence of Cdk2 activity, and cause centrosome re-duplication [31] in all human cells thus far tested [47], even at expression levels that are roughly two-fold lower than that of endogenous hMps1 [47]. Accordingly, in human cells the failure of wild type hMps1 to cause centrosome re-duplication appears to be the consequence of its efficient proteasome-dependent removal from centrosomes, but preventing this removal is sufficient to cause centrosome re-duplication [29,31,47]. Moreover, while cyclin A-dependent centrosome re-duplication requires hMps1, that caused by hMps1^Δ12/13^, hMps1^T468D^, or hMps1^T468E ^no longer requires cyclin A [31], suggesting that cyclin A promotes centrosome re-duplication by suppressing the degradation of hMps1 at centrosomes.

The observation that very modest changes in centrosomal hMps1 can cause centrosome re-duplication suggests that defects in the control of hMps1 degradation might contribute to centrosome abnormalities in human tumors. In fact, hMps1 is not appropriately degraded in response to the Cdk2 inhibitor roscovitine in a variety of tumor-derived cells [47]. Transgenic hMps1 can be degraded appropriately in one such cell type, the U2OS osteosarcoma cell line well known to undergo centrosome re-duplication, suggesting that at least in these cells the defect is intrinsic to endogenous hMps1. While no hMPS1 coding mutations were found in U2OS cells, the hMPS1^Δ12/13 ^mRNA lacking exons 12 and 13, which generates the internally truncated hMps1^Δ12/13 ^protein lacking amino acids 420-507 discussed above, was cloned by RT-PCR amplification of hMPS1 mRNAs from U2OS cells [47]. However, the 21NT mammary carcinoma cell line fails to degrade both endogenous and transgenic hMps1, and GFP-hMps1^T468A ^can accelerate centrosome re-duplication in 21NT cells, suggesting that defects in hMps1 degradation can also be caused by deficiencies in the factors that regulate its degradation [47]. In fact, we have recently shown that hMps1 degradation is controlled by a potential tumor suppressor, Ornithine Decarboxylase Antizyme (OAZ, or Antizyme) [29]. Together, these studies demonstrate that cells normally exercise exquisite control over centrosomal Mps1, and that even modest changes in the centrosomal pool of Mps1 are sufficient to break the control of centrosome duplication to generate excess centrioles.

### What is the MDS?

The MDS contains no known targeting motifs for either the Skp/Cullin/F-box (SCF) or Anaphase Promoting Complex/Cyclosome (APC/C) E3-ubiquitin ligases, and its only obvious similarity is to other vertebrate Mps1 proteins [31], suggesting that the MDS controls centrosomal Mps1 through a mechanism independent of typical SCF or APC/C degradation pathways. OAZ is a potential tumor suppressor that targets a handful of known substrates for ubiquitin-independent proteasome-mediated degradation [71]. For example, OAZ regulates polyamine biosynthesis by binding to and targeting Ornithine decarboxylase to the proteasome for degradation in a ubiquitin-independent manner [72]. Mangold et al. showed that OAZ and its inhibitor (Antizyme Inhibitor, or AZI) are found at centrosomes, and that OAZ activity suppresses centrosome re-duplication; increased OAZ activity and/or levels suppressed centrosome re-duplication, while reducing OAZ led to centrosome amplification [73]. Based on this data Mangold et al. hypothesized that OAZ promoted the degradation of a centrosomal substrate whose continued presence at centrosomes promoted centrosome re-duplication [73]. The phenotypes described by Mangold et al. could be easily explained if that substrate were hMps1, making OAZ a very attractive candidate for mediating the MDS-dependent removal of hMps1 from centrosomes. Indeed, OAZ binds to hMps1 through the MDS (Figure 2), regulating centrosomal hMps1 levels in a proteasome-dependent manner, and antagonizing hMps1-dependent centrosome re-duplication in a manner that is attenuated by T468 phosphorylation [29].

### Is the MDS the only control over centrosomal Mps1?

Given the multiple functions of Mps1, it seems likely that it might be controlled by multiple mechanisms. Indeed, yeast Mps1 has been shown to be a substrate of the APC/C, and mutual antagonism between Mps1 and the APC/C is of central importance to proper activation and silencing of the SAC in yeast [74]. Moreover, a recent study by Cui et al. has shown that hMps1 is a ubiquitinated protein, and that both Cdc20 and Cdh1 promote the APC/C-dependent degradation of hMps1 during mitosis and G1, respectively [75]. Interestingly, Cui et al. found that mutation of the single D-box present in the N-terminus of hMps1 (amino acids 256-263, see Figure 2) not only led to increased hMps1 levels, but also caused centrosome amplification [75]. While they did not characterize the mechanism of centrosome amplification, it is tempting to speculate that increasing the cytoplasmic pool of hMps1 indirectly increased the centrosomal pool as well, leading to centrosome re-duplication. Together, this suggests that two mechanisms cooperate to control the function of hMps1 in centrosome duplication (Figure 2); first, APC/C-mediated proteolysis acts on hMps1 through its D-box as cells exit mitosis and enter G1 in order to reduce the cytoplasmic pool of hMps1 [75] (which is roughly 5-10 fold higher during the SAC [64]), and second, OAZ-mediated proteolysis acts on hMps1 through its MDS to keep centrosomal hMps1 levels low until Cdk2 activity is sufficiently high to launch the early events of centrosome duplication. Once centrosomes have duplicated, OAZ again acts through the MDS to reduce centrosomal levels and suppress centrosome re-duplication [29,73].

## IV. Recent studies identify multiple centrosomal functions for hMps1

### OAZ control of Mps1 reveals a role for Mps1 in procentriole assembly

**Figure 3:**
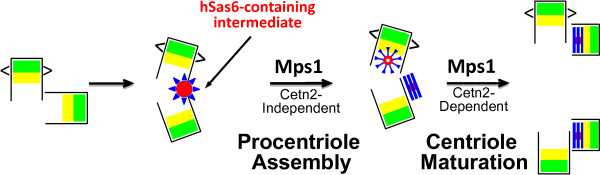
**Proposed functions for Mps1 in centriole assembly**. Studies with OAZ and Cetn2 suggest two functions for Mps1 in centriole assembly, an early function in procentriole assembly and a later function in centriole maturation that is mediated by Cetn2. We propose that the single centriolar focus of hSas6 observed upon overexpression of OAZ or hMps1KD represents an intermediate in centriole assembly (depicted as a red and blue sunburst, red indicating hSas6 and blue representing other cartwheel proteins). The observation that this structure is observed upon attenuation of Mps1 activity [29] suggests that Mps1 activity remodels this intermediate into cartwheels, onto which procentrioles are subsequently assembled. Because hMps1^Δ12/13 ^generates excess hSas6-containing structures in the absence of Cetn2 [28], this early function of Mps1 is presumably Cetn2-independent. The observation that Mps1 is required for the ability of Cetn2 to organize distal centriole elements [28] suggests that Mps1 has a second, Cetn2-dependent function in the maturation of centrioles. Structures and colors are as in Figure 1, with the exception of carets used to depict the centriolar appendages of the maternal centriole, and the sunburst depicting the hSas6-containing intermediate.

The existence of such exquisite control over centrosomal hMps1 and the consequences of subtle changes in this control make it tempting to speculate that hMps1 must play some important role at centrosomes, and indeed two recent studies have revealed at least two functions for hMps1 in centrosome duplication. First, our study documenting the role of OAZ in removing hMps1 from centrosomes revealed a role for Mps1 early in centriole assembly [29]. The majority of experiments in that study explored the ability of OAZ to suppress the function of hMps1 in centrosome re-duplication; the binding of OAZ to hMps1 promotes the MDS- and proteasome-dependent removal of hMps1 from centrosomes, preventing it from accelerating centrosome re-duplication [29]. However, we also found that OAZ antagonized the canonical centrosome duplication cycle. In a G1 enrichment and release assay, overexpression of OAZ led to a five-fold increase in the number of S-phase HeLa cells with just two centrioles [29], and the majority of these had a single focus of hSas6 staining [29]. As discussed above, cells normally have zero or two hSas6 foci. Accordingly, the observation that OAZ-overexpressing cells with two centrioles have one hSas6 focus supports a role for hMps1 early in procentriole assembly. Both the increase of cells with two centrioles and the hSas6 assembly phenotype appear to be due to attenuation of hMps1, because both were complemented by overexpression of wild type hMps1, and both were phenocopied by overexpression of hMps1KD [29]. OAZ overexpression likely causes a delay in assembly rather than a block, because the number of S-phase cells overexpressing OAZ that have two centrioles was markedly lower in asynchronous cultures that had not been previously enriched in G1 [29]. Thus, our examination of the control of centrosomal hMps1 levels led to the demonstration that hMps1 functions very early in centriole assembly, and suggests that hMps1 phosphorylation promotes the remodeling of an hSas6-containing intermediate into procentrioles (Figure 3). This proposed function for hMps1 is similar to the function of budding yeast Mps1p in SPB assembly. Mps1 phosphorylation of Spc42p remodels overexpressed Spc42p from a spherical structure adjacent to the SPB into an planar extension of the SPB central plaque [76]. Similarly, Mps1p phosphorylation was found to modulate complexes formed by Cdc31p (the yeast centrin orthologue) and Spc29p, suppressing the interaction between Cdc31p and Kar1p to promote satellite assembly, and enhancing the interaction between Spc29p and Bbp1p to promote the subsequent insertion of the nascent SPB into the nuclear envelope [77].

### Analysis of the Mps1 substrate Cetn2 reveals multiple Mps1 functions

The observation that non-degradable hMps1 proteins cause centriole overproduction [31] even at very modest expression levels [47] suggests that sustained phosphorylation of some set of centriole proteins by hMps1 leads to excess centriole assembly. A second study identifying the centriolar protein Cetn2 as one such hMps1 substrate provided additional evidence for an early function for hMps1, and also suggested at least one additional function for hMps1 in centriole assembly [28]. Three *in vitro *sites of hMps1 phosphorylation were identified in Cetn2, and each was shown to be important for the control of centriole number (although *in vivo *phosphorylation at these sites was not demonstrated) [28]. Interestingly, a role for Cetn2 in centriole assembly has also been controversial. The first siRNA-mediated depletion of Cetn2 suggested that it was essential for centriole assembly in HeLa cells, and while there was a dearth of centriolar markers available for analysis at the time, electron microscopy showed an increase of cells with fewer than expected centrioles [33]. In contrast, subsequent studies found that co-depletion of Cetn2 and Cetn3 had no affect on Plk4-mediated centriole overproduction [15], and that depletion of Cetn2 alone had no affect on recruitment of hSas6 to centrioles [14]. Our experiments support the suggestion that while hMps1 phosphorylation sites in Cetn2 enhance the rate of centriole assembly, at least in HeLa cells Cetn2 is dispensable for the canonical centriole assembly pathway [28]. However, the presence of Cetn2 increases the rate of centriole assembly (as judged by CP110 recruitment), and this function of Cetn2 requires the major site of *in vitro *hMps1 phosphorylation, T118 [28]. Moreover, Cetn2 is required for the maturation of excess centrioles produced in cells expressing hMps1^Δ12/13 ^[28]. Interestingly, we found that even in the absence of Cetn2 expression of hMps1^Δ12/13 ^led to the assembly of excess hSas6 containing structures, but that these structures could not recruit pericentriolar material in the absence of Cetn2 [28]. The observation that attenuation of hMps1 retards hSas6 assembly in the canonical centriole assembly cycle [29], while hMps1^Δ12/13 ^promotes the assembly of excess hSas6 containing structures [28] further supports the suggested role for hMps1 in procentriole assembly. Moreover, this data also suggests that Cetn2 cannot be the only Mps1 substrate whose phosphorylation is critical for centriole overproduction. Regardless, taken together the OAZ and Cetn2 studies suggest that Mps1 has at least two functions in centriole assembly (Figure 3). In one function, mediated by as yet unidentified substrate(s), hMps1 promotes the remodeling of a single hSas6-containing precursor into cartwheels adjacent to each existing centriole. In a second function, hMps1 promotes the Cetn2-dependent maturation of the resulting procentrioles.

**Figure 4:**
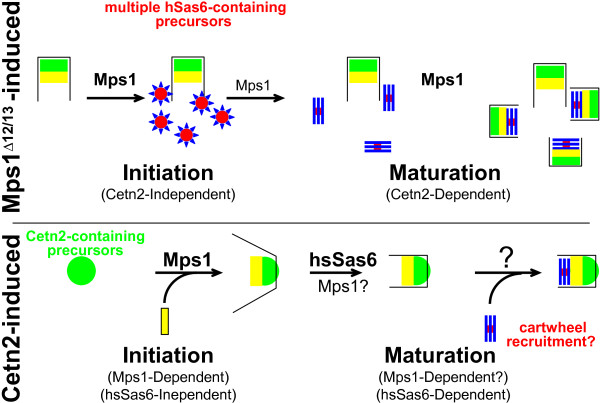
**Modes of centriole over production**. We propose that hMps1 and Cetn2 cooperate to generate centriole overproduction by two distinct mechanisms. First, in Mps1^Δ12/13^-induced centriole overproduction, hMps1^Δ12/13 ^promotes the assembly of multiple hSas6 containing precursors (analogous to the proposed hSas6-containing intermediate in procentriole assembly described in Figure 3). As in the canonical centriole assembly pathway, we assume that hMps1 is required for the remodeling of these precursors into cartwheels, onto which procentrioles are assembled. The figure reflects the possibility that not all of the precursors become cartwheels, based on the observation that the percentage of cells with excess hSas6 foci is greater than the percentage of cells with excess γ-Tubulin or CP110 foci [28]. Second, hMps1 is also required for initiation of Cetn2-induced centriole overproduction. We propose that overexpression of wild type Cetn2 leads to the assembly of Cetn2-containing precursors that organize distal centriole modules in a top-down fashion that is independent of hSas6. Because Mps1 is required for the initiation of these structures, we cannot assess a role for Mps1 in their maturation. However, while the initiation of these structures is hSas6-independent, some aspects of their maturation require hSas6, as suggested by the observation that a subset of centriole proteins are not recruited to these structures in the absence of hSas6 [28]. Because these structures recruit hSas6, it is possible that they can recruit cartwheels (indicated by a question mark). Structures and colors are as in Figure 3.

Interestingly, our study of Cetn2 also suggests that Cetn2 has multiple roles in centriole assembly. Mutations that mimic phosphorylation at any of the three hMps1 phosphorylation sites, e.g. Cetn2^T118D^, led to a robust overproduction of *bona fide *centrioles [28]. The ability of phosphomimetic Cetn2 mutants to promote centriole overproduction required hSas6 [28], suggesting that hMps1 phosphorylation sites within Cetn2 stimulate centriole overproduction through cartwheel templates. Centriole overproduction in cells expressing phosphomimetic Cetn2 mutants also required hMps1 [28], further supporting the suggestion that additional centriolar hMps1 substrates must exist. Interestingly, we found that overexpression of wild type Cetn2 led to the overproduction of both *bona fide *centrioles and aberrant centriole-like structures [28] that required hMps1 but not hSas6, suggesting that wild type Cetn2 did not lead to centriole overproduction through a cartwheel template. These observations suggest that Mps1 and Cetn2 cooperate to promote centriole overproduction via multiple mechanisms (Figure 4). First, expression of the non-degradable hMps1^Δ12/13 ^induces the typical "Bottom-up" production of excess centrioles via hSas6-containing precursors. The initiation step does not require Cetn2, but is followed by the Cetn2-dependent maturation of these structures into centrioles. Although it remains to be tested, this pathway is presumably similar to that induced by overexpression of Plk4 or hSas6. Second, hMps1 is also required for the production of excess centriole-like structures induced by overexpression of wild type Cetn2. Because it does not require hSas6, the assembly of these structures is presumably initiated from Cetn2-containing precursors that first organize distal centriole elements, and then recruit proximal elements such as Cep135 and hSas6 in a "Top-down" fashion. Because these Cetn2-induced structures can recruit hSas6, it seems possible that they may contain cartwheels, although their initiation is not cartwheel dependent. While the initiation step in Cetn2-induced centriole overproduction does not require hSas6, some aspects of the maturation of these structures is hSas6-dependent; in the absence of hSas6 these structures can no longer recruit proximal centriole elements like Cep135, although they can still recruit pericentriolar material and function as mitotic spindle poles [28]. Together, these studies on OAZ and Cetn2 support the suggestion that hMps1 has both an early role in procentriole assembly, and a later Cetn2-dependent role in centriole maturation. Consistent with findings from yeast [76-78], our studies suggest that hMps1 phosphorylation of centriolar protein(s) remodels protein complexes to promote initiation and maturation of procentrioles, and that this is relevant to both the canonical centriole assembly pathway and centriole overproduction.

## V. But is Mps1 essential?

### Mps1 inhibition

Despite observations with Cetn2 and OAZ that suggest centrosomal functions for hMps1, several recent studies using chemical genetics [79-81] or small molecule inhibitors [60,82-84] have lent support to the suggestion that hMps1 is not essential for centrosome duplication. In two of the chemical genetics studies, cell lines that depend solely on an analog-sensitive (as) version of hMps1 (hMps1-as, engineered to be sensitive to inhibition by bulky ATP analogs) were constructed by expressing an RNAi-resistant version of hMps1-as together with an hMps1-specific shRNA [80,81]. In a third chemical genetics study hMps1-as was expressed using retroviral vectors in cells harboring a deletion at the endogenous hMps1 locus constructed using Adeno-Associated Viral vectors [79]. Although two of these studies did not mention centrosomes, Tighe et al. comment that they observed no centrosomal defects [81]. Several small molecule inhibitors of hMps1 have also been reported, and while they all perturb mitosis, those studies that examined centrosomes similarly reported that hMps1 inhibition had no consequence for centrosome duplication [60,83,84].

**Figure 5:**
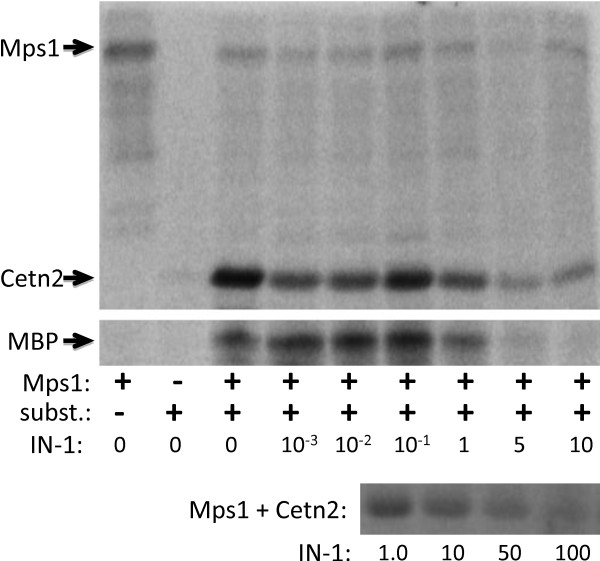
**IN-1 is not equally effective against all Mps1 substrates *in vitro***. *In vitro *kinase assays were performed as described by Yang et al. (2010) [28], with GST-Mps1 (0.4 mM) and either 6hisCetn2 (Cetn2) or Myelin Basic Protein (MBP) as substrate (10 mM). The IN-1 Mps1 inhibitor described by Kwiatkowski et al. (2010) [60] was included at the indicated concentrations in μM. The top and bottom panels show autoradiographs of kinase assays with Cetn2 as substrate (subst.), the bottom cropped to show just Cetn2. The middle panel shows a similar kinase assay using MBP as substrate, cropped to show just MBP. Arrows to the left indicate the signals corresponding to Mps1 autophosphorylation (which is attenuated in the presence of Cetn2), Cetn2 phosphorylation, and MBP phosphorylation. While enzyme and substrate concentrations differ from those used by Kwiatkowski et al. (2010) [60], Cetn2 phosphorylation and Mps1 autophosphorylation were observed at IN-1 concentrations that blocked MBP phosphorylation (5 and 10 μM IN-1), and residual Cetn2 phosphorylation was observed even at 100 μM IN-1.

There are of course caveats with both chemical genetics and kinase inhibitors. First, as discussed above, partial depletion of hMps1 causes mitotic catastrophe despite the presence of residual hMps1 [32], and it seems likely that a partial inhibition of hMps1 would produce the same effects. Indeed, the zebrafish nightcap mutation was shown to be a hypomorphic allele of Mps1 that caused mitotic defects despite the presence of residual Mps1 activity [54] (although a null allele ultimately suggested that Mps1 was not required for centrosome duplication in zebrafish [85]). Given that partial depletion [32] or inhibition [54] of Mps1 can cause such severe mitotic defects, it becomes difficult to know when depletion/inhibition is complete. Second, as we demonstrated for hMps1 siRNA, cells with reduced hMps1 undergo mitotic and cytokinetic failures that produce G1 cells with two centrosomes, potentially masking subsequent defects in centrosome duplication [32]; we suspect that this would be a greater issue for the acute inhibition of hMps1 than for the gradual rundown of hMps1 levels achieved using RNAi. Third, unlike RNAi experiments, the presence of the inhibited kinase might allow hMps1 to participate in non-enzymatic functions; that is, accepting the conclusions from chemical genetics and small molecule studies, they tell us that hMps1 kinase activity is dispensable, but do not demonstrate that the hMps1 protein is dispensable. Small molecule inhibitors have the additional caveat that they may not be equally effective against all substrates, and this appears to be the case for the IN-1 inhibitor, which we obtained as a very gracious gift from Dr. Nathaniel Gray (Dana-Farber Cancer Institute, Boston MA). At 10 μM IN-1 abolishes the *in vitro *phosphorylation of MBP by hMps1 (Figure 5) and causes mitotic defects *in vivo *[60], but it requires significantly higher concentrations of IN-1 to completely block *in vitro *Cetn2 phosphorylation (Figure 5). While this *in vitro *result was obtained under different conditions than used by Kwiatkowski et al. [60] and cannot be directly compared to the use of IN-1 *in vivo*, it supports the possibility that despite causing mitotic failures, hMps1 inhibitors might leave sufficient residual hMps1 activity to support centrosome duplication.

### Functional studies

Regardless, there is now a preponderance of evidence to suggest that hMps1 is dispensable for centriole assembly. Even some aspects of our own data on Cetn2 support this suggestion. Although hMps1 has Cetn2-independent functions in centriole assembly [28], the identification of a non-essential substrate for hMps1 suggests that at least one of its functions might be dispensable. Moreover, the data we used to conclude that Cetn2 is dispensable for centriole assembly [28] was very similar to that used to reach the opposite conclusion for hMps1 [32]. In our first study of hMps1, we assessed centriole number as HeLa cells that had been enriched in G1 by serum starvation entered S-phase after re-addition of serum (HeLa cells do not arrest in response to serum starvation, but rather grow slowly and accumulate in G1). Using a single time point eight hours after the addition of serum and 5-bromo-2'-deoxyuridine (BrdU), we observed that hMps1 depletion led to a large increase in the percentage of cells that had entered S-phase during the release period but had just two centrioles, and concluded that hMps1 was required for centrosome duplication [32]. In our Cetn2 study we measured centriole number in BrdU positive cells form unstarved HeLa cultures after a 4 hr BrdU pulse, and found that Cetn2-depletion caused a two-fold increase in the number of S-phase cells that had just two centrioles [28]. However, we also included a chase period in order to determine the fate of the centrioles that had not duplicated during the initial BrdU pulse. We found that after a four hour chase period the percentage of Cetn2-depleted cells with two centrioles dropped to that seen in controls [28], suggesting that centriole assembly is initially delayed in the absence of Cetn2, but is ultimately completed. While there are important differences between these two approaches (starved vs unstarved cells, long labeling vs pulse chase), they are similar enough to prompt us to consider whether our original data with hMps1 might also reflect a delay. Accordingly, it still remains unclear whether hMps1 is essential for centriole assembly, and it will ultimately require a detailed comparison of the depletion/inhibition of hMps1 with the depletion of other essential (e.g. Plk4) and non-essential factors (e.g. Cetn2) under identical experimental conditions to resolve the issue.

### Do we even care if hMps1 is essential?

If hMps1 has no role in centrosome duplication, its conservation is somewhat hard to explain, particularly given that phosphorylation of centrins is conserved down the actual phosphorylation site; yeast Mps1p phosphorylates Cdc31p at T110 [77], which is analogous to the T118 residue found to be the major hMps1 phosphorylation site within Cetn2 [28]. If hMps1 has no role in centrosome duplication it is also hard to understand why cells would exert so much control over the centrosomal levels of hMps1, and more importantly why subtle changes in centrosomal hMps1 levels would lead to centrosome re-duplication [28,29,31,47]. Therefore, it seems clear that hMps1 plays some role in centriole assembly, regardless of whether it is essential for the process. Perhaps hMps1 takes on different importance in different cellular contexts. Not only did our recent work show that Centrins 1, 2, and 3 are not functionally equivalent, but it also showed that Cetn2 has cell type specific effects [28]. Perhaps hMps1 has similar cell type specificity, and is not essential for centriole assembly in the commonly used cell lines. Cep76 also exhibits cell type specific function [35], suggesting that the requirements for centriole assembly may vary considerably according to cell type. It is also possible that hMps1 plays a more important role at some point in development, but that tumor cells re-activate multiple pathways for centriole assembly such that it becomes dispensable.

Another possibility is that Mps1 may regulate the fidelity of centriole assembly or transmission. Depletion of the *T. thermophila *TtCEN1 leads to the progressive loss of basal bodies (structures analogous to centrioles), and those that remain are deteriorated, suggesting that TtCEN1 is required not just for assembly but also for the maintenance of basal bodies [86]. A similar phenotype was seen in PtCEN2-depleted *P. tetraurelia*, but PtCEN2 was demonstrated to have no overt role in either the initiation or assembly of new basal bodies [87]. Rather, PtCEN2 was shown to regulate the selection of the site and orientation of basal body assembly as well the stability of basal bodies, both of which are critical for proper basal body retention and function [87,88]. Our data suggest that Cetn2 increases the rate of centriole assembly, and given the requirement for hMps1 in Cetn2 function [28], perhaps hMps1 is also required for aspects of site selection and maintenance that are dispensable for the actual assembly of centrioles.

### An Interesting Twist

Inhibition of hMps1 via chemical genetics and small molecules suggests that it is essential in vertebrates [60,79-84], and consensus is that its essential function is in the SAC [55]. It is interesting to note that in yeast the SAC itself is not essential, and so represents the dispensable function of yeast Mps1p. In contrast, in vertebrates the SAC is essential and centrosomes themselves are dispensable (at least for the progression of mitosis) [89]. If Mps1 is dispensable for centrosome duplication in vertebrates, perhaps this is because vertebrates evolved to rely on it for the spindle checkpoint, which presumably becomes increasingly important with increasing genomic complexity, and thus evolved mechanisms to replace its function in centrosome duplication.

## Conclusions

We may ultimately find that hMps1 is dispensable for centriole assembly. However, that would not dampen our interest in Mps1, and would still leave it in good company. Cdk2 was long assumed to be essential not just for centrosome duplication but for DNA replication as well, yet mice null for Cdk2 are viable [90] and Cdk2 is dispensable for centrosome duplication [91]. This doesn't mean that Cdk2 is unimportant, and indeed Cdk2 is required for centrosome re-duplication [91]. Likewise, Cetn2, perhaps the most well known centriole marker, is dispensable for centriole assembly [14,15,28]. While it is clearly necessary to understand the core evolutionarily conserved centriole assembly pathway, it is also critical to understand how centriole assembly occurs within a human cell when all the components are present. It is also critical to understand the types of defects that might contribute to the appearance of extra centrosomes in tumors, even if those defects involve players that are not essential for the canonical assembly cycle such as Cdk2 [91] and Cetn2 [28]. Indeed, centriole overproduction has proven a useful model system for understanding centriole assembly in general [15]. Regardless of whether centrioles can be assembled in the absence of hMps1, our recent data support the idea that when present it plays multiple roles in centriole assembly. Indeed, even this seems to be conserved from yeast, where Mps1 acts in at least two steps in SPB duplication [78]. Moreover, subtle changes in hMps1 or its regulators can lead to centriole overproduction [28,29,31,47], and although it remains to be demonstrated we presume that this reflects the failure to properly control its functions in the canonical centriole assembly cycle, even if those functions are dispensable.

**Figure 6:**
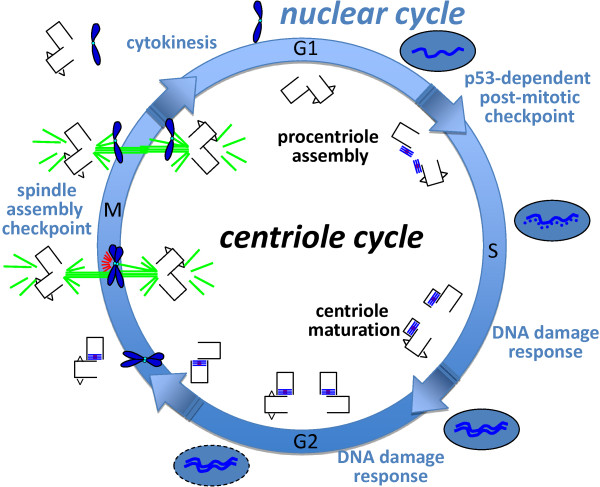
**The many roles of Mps1 in the maintenance of genomic integrity**. Many functions for hMps1 have been described in human cells, in both the centriole and nuclear cycles. Although the described functions for hMps1 are quite varied, when properly coordinated they ensure the integrity of the genome. Recent work discussed extensively in this review suggests that hMps1 functions in both procentriole assembly and centriole maturation. While it is possible that these functions are dispensable, failure to properly regulate them leads to centriole overproduction that can generate aberrant mitotic spindles. hMps1 has also been implicated in cytokinesis [59], responsible for partitioning of one copy each of the duplicated genome and centrosome into daughter cells. hMps1 regulates the p53-dependent post-mitotic checkpoint that prevents cell cycle entry after failed mitosis, and defects in this function can allow aneuploid cells to proliferate [57]. hMps1 regulates the Chk2-dependent DNA damage response, and defects in this function lead to defective arrest in the presence of damaged DNA [58]. Finally, hMps1 regulates the spindle assembly checkpoint [55], and defects in this function lead to chromosome segregation errors. The function of hMps1 in SMAD signaling represents a transcriptional input into the spindle assembly checkpoint [56]. In this figure, a circle represents the cell cycle, with phases labeled and arrows representing transitions. The centriole cycle is depicted on the inside, with carets indicating centriolar appendages (which are assembled onto the two oldest centrioles during mitosis) and cartwheels (which are degraded during mitosis) as in Figures 1, 3, and 4. The nuclear cycle is depicted on the outside, with the nucleus as a grey oval, and chromosomes in blue. The mitotic spindle is indicated by green lines representing microtubules, mitotic chromosomes in blue, kinetochores in cyan, and red lines indicating an activated spindle checkpoint.

Mps1 is an intriguing multifunctional enzyme with multiple roles in both the centriole and nuclear cycles (Figure 6). In the centriole cycle these functions include its roles in procentriole assembly and centriole maturation, discussed above, critical for bipolar mitotic spindle assembly and accurate segregation of the duplicated genome. In the nuclear cycle these functions include the SAC that ensures proper segregation of the duplicated genome, cytokinesis that ensures the final partitioning of the duplicated genome, and at least two different cellular responses that halt cell cycle progression in the presence of missegregated or damaged DNA. Accordingly, each of the functions thus far described for Mps1 in human cells is related to the maintenance of genomic integrity. While these Mps1 functions may be of differing importance in different contexts, it will be critical to understand how each of these functions is regulated given that hMps1 has been proposed as a target for cancer therapy (see [55] and references therein). Clearly, there are many challenges ahead for understanding the role of hMps1 at centrosomes. First, we must determine one way or the other whether it is essential for centriole assembly. However, regardless of the answer, we must take a more systematic approach to identifying the centrosomal substrates of hMps1, in order to characterize the role of hMps1 phosphorylation, dispensable or otherwise, in centriole assembly at a molecular level. In addition, we need to place hMps1 in the great puzzle that is centriole assembly. While Plk4 is the major driver of centriole assembly, it is not functionally interchangeable with Zyg-1 [92], nor is it apparently a true orthologue of Zyg-1, which according to a recent study is more closely related to Mps1 [20,92]. Moreover, while in *C. elegans *the recruitment of Sas-6 to the procentriole requires ZYG-1 [7,8], Plk4 is not required for recruitment of hSas6 to the centriole, although it is required for the maintenance of hSas6 at the centriole and for daughter centriole assembly [14]. This suggests that Plk4 does not assume all of the functions ascribed to ZYG-1, and that other players must be involved in human cells. We assume that hMps1 is one of these players, and a future challenge will be to determine the relationships between hMps1, Plk4, and hSas6. Moreover, Cetn2 has as yet unidentified functions in centriole assembly that are independent of hSas6 but require hMps1, suggesting that hMps1 might also have centrosomal functions outside of the canonical cartwheel driven centriole assembly pathway.

## List of Abbreviations Used

Cetn2: Centrin 2; SPB: spindle pole body; Mps1: Monopolar spindles 1; SAC: Spindle Assembly Checkpoint; mMps1: mouse Mps1; hMps1: human Mps1; KD: kinase dead; Cdk2/A: Cyclin A-associated cyclin-dependent kinase 2; MDS: Mps1 degradation signal; OAZ: Ornithine Decarboxylase Antizyme; SCF: Skp/Culling/F-box; APC/C: anaphase promoting complex/cyclosome; as: analog-sensitive; BrdU: 5-bromo-2'deoxyuridine.

## Competing interests

The authors declare that they have no competing interests.

## Authors' contributions

ANP and HAF made equal contributions to construction of the manuscript. ANP performed the kinase assays in Figure 5, and HAF constructed the figures. Both authors read and approved the final manuscript
